# Correlation between sequence divergence and polymorphism reveals similar evolutionary mechanisms acting across multiple timescales in a rapidly evolving plastid genome

**DOI:** 10.1186/s12862-014-0268-y

**Published:** 2014-12-24

**Authors:** Karen B Barnard-Kubow, Daniel B Sloan, Laura F Galloway

**Affiliations:** Department of Biology, University of Virginia, P.O. Box 400328, Charlottesville, VA 22904-4328 USA; Department of Biology, Colorado State University, Fort Collins, CO 80523 USA

**Keywords:** Plastid, Selection, dN/dS, pN/pS, Reproductive isolation, Intraspecific, Chloroplast, Sequence evolution

## Abstract

**Background:**

Although the plastid genome is highly conserved across most angiosperms, multiple lineages have increased rates of structural rearrangement and nucleotide substitution. These lineages exhibit an excess of nonsynonymous substitutions (i.e., elevated dN/dS ratios) in similar subsets of plastid genes, suggesting that similar mechanisms may be leading to relaxed and/or positive selection on these genes. However, little is known regarding whether these mechanisms continue to shape sequence diversity at the intraspecific level.

**Results:**

We examined patterns of interspecific divergence and intraspecific polymorphism in the plastid genome of *Campanulastrum americanum*, and across plastid genes found a significant correlation between dN/dS and pN/pS (i.e., the within-species equivalent of dN/dS). A number of genes including *ycf1*, *ycf2*, *clpP*, and ribosomal protein genes exhibited high dN/dS ratios. McDonald-Kreitman tests detected little evidence for positive selection acting on these genes, likely due to the presence of substantial intraspecific divergence.

**Conclusions:**

These results suggest that mechanisms leading to increased nucleotide substitution rates in the plastid genome are continuing to act at the intraspecific level. Accelerated plastid genome evolution may increase the likelihood of intraspecific cytonuclear genetic incompatibilities, and thereby contribute to the early stages of the speciation process.

**Electronic supplementary material:**

The online version of this article (doi:10.1186/s12862-014-0268-y) contains supplementary material, which is available to authorized users.

## Background

The structure of the plastid genome is generally conserved across the angiosperms [1], and its nucleotide substitution rates are usually low relative to the nuclear genome [2]. However, multiple lineages exhibit extensively rearranged plastid genomes as well as increased rates of nucleotide substitution and elevated dN/dS ratios for some genes [3]. While this connection suggests the potential for a common underlying cause of structural instability and increased nucleotide substitution rates [3], it is important to note that while structural instability impacts the plastid genome as whole, the increase in substitution rate appears to vary depending on gene function [4]. In lineages that show increased substitution rates, similar sets of plastid genes have experienced increased substitution rates and elevated dN/dS ratios, suggesting the possibility of common mechanisms or selective regimes acting on these genes in independent angiosperm groups. However, it remains unclear whether these patterns reflect positive selection, relaxed purifying selection, changes in underlying mutation rates, a breakdown in DNA repair mechanisms such as gene conversion [5], or some combination of these.

Accelerated evolution in the plastid genome of some angiosperm lineages raises the question as to whether the mechanisms responsible continue to operate at the intraspecific level. Numerous studies examining nucleotide substitution rates and potential signatures of selection in the plastid genome, based on variation in dN/dS, have been carried out based on divergence among species [4,6-10], but data describing genome-wide intraspecific sequence variation are needed to investigate whether accelerated plastid genome evolution is occurring within species. Estimates of intraspecific polymorphism are also useful for interpreting interspecific divergence, as it allows for distinguishing between the effects of positive selection and relaxed purifying selection on nucleotide substitution rates based on changes in the relative ratio of non-synonymous to synonymous changes before and after selection has acted [11,12]. Accordingly, positive selection is expected to lead to a significantly higher ratio for interspecific divergence than for intraspecific polymorphism.

We examined patterns of sequence divergence and polymorphism in *Campanulastrum americanum* to determine whether similar mechanisms of plastid genome evolution are acting within as well as between species. This species is a good study system in which to address these questions as it is in the Campanulaceae, a family in which the taxa have highly rearranged plastid genomes [13,14], increasing the likelihood of detecting intraspecific accelerated plastid evolution. In particular we sought to answer the following questions. 1) Do a similar set of plastid genes exhibit increased nucleotide substitution rates and elevated dN/dS ratios in *C. americanum* as found in previous studies with other species? 2) Are similar patterns found when examining plastid sequence variation among populations within *C. americanum*? 3) Do we find evidence for positive selection leading to increased substitution rates and elevated dN/dS ratios in these plastid genes?

## Methods

### Study system

*Campanulastrum americanum* is a monocarpic herb found in the eastern half of the United States. Individuals are autotetraploid, annual or biennial, and primarily outcrossing [15,16]. The Campanulaceae has been shown to have highly rearranged plastid genomes as well as the potential for biparental plastid inheritance [17,18]. Crossing studies in *C. americanum* have found that while inheritance is primarily maternal, biparental and paternal inheritance occurs in roughly 25% of offspring (Barnard-Kubow, unpublished results). However, plastid polymorphism within populations appears relatively low, with genotyping at five loci (including portions of ycf1, rps2, and rps4) finding individuals within a population to be generally fixed for plastid haplotype (Barnard-Kubow, unpublished results). Therefore, while biparental inheritance may complicate full assembly of the plastid genome when using a maternal family, it is unlikely to cause significant error in terms of estimates of polymorphism or in determining the presence/absence of plastid genes.

### Sample material and library construction

For sequencing the plastid genome of *C. americanum*, 180 grams of fresh leaf tissue was collected from multiple individuals from a single maternal family (i.e., seeds from a single plant) from a population in Virginia (Table 1). Individuals were germinated in a growth chamber from field-collected seed and grown for several months in the greenhouse with regular watering and fertilization.

**Table 1:** **Location information for the populations used in this study**

**Population**	**Latitude**	**Longitude**
VA (Virginia, USA)	37.35495	−80.55415
AL (Alabama, USA)	34.65048	−86.51643
MN (Minnesota, USA)	44.81650	−93.30758
OH (Ohio, USA)	41.11472	−81.51806

For examining within species polymorphism of the plastid genome, 150 grams of fresh leaf tissue was collected and pooled from multiple individuals from four populations of *C. americanum* (VA, MN, OH, and AL), including the same VA population used for the single population plastid sequencing (Table 1). These populations were chosen because they span the geographic range of *C. americanum* and were known to differ genetically based on sequencing of individual chloroplast loci (Barnard-Kubow et al., unpublished results). VA individuals were transplanted from the field, while MN, OH, and AL individuals were germinated from field-collected seed in a growth chamber. Plants from all four populations were then grown for several months in the greenhouse with regular watering and fertilization. Intact chloroplasts were isolated from the single population (VA) and pooled samples using a combination of differential centrifugation and separation on a sucrose step gradient [19,20]. Chloroplasts were then lysed, and DNA was obtained via a phenol-chloroform extraction and ethanol precipitation. The purity of plastid DNA (cpDNA) was confirmed by restriction digestion.

For each plastid sample, shotgun libraries were constructed with multiplex identifier (MID) tags following standard protocols for sequencing on a Roche 454 GS-FLX platform with Titanium reagents. MID-tagged libraries were sequenced as part of a larger pooled sample. All 454 library construction and sequencing steps were performed at the Genomics Core Facility in the University of Virginia’s Department of Biology. A total of 28,694 and 24,552 sequencing reads were obtained from the VA and pooled libraries, respectively. The mean sequence lengths were 335 bp for the VA library and 339 bp for the pooled library. The reads from each library were deposited in NCBI’s Short Read Archive [SRX595708 and SRX595709].

### Plastid assembly and annotation

454 reads for the single population sample were assembled using Roche’s GS de novo Assembler v2.3 (“Newbler”) using default settings. Initial assembly produced hundred of contigs, however many of these were identified as bacterial or nuclear contamination. By visualizing the remaining contigs in Consed v21 [21] and using information regarding reads that span multiple contigs, 63 of the initial contigs were re-assembled into nine final contigs with a total length of 147.3 kb and an average single copy coverage depth of 20×. For the *ccsA* gene, PCR and Sanger sequencing were used to obtain sequence spanning a gap and complete the full sequence.

DOGMA [22] was used to annotate the protein, transfer RNA (tRNA), and ribosomal RNA (rRNA) genes for each of the contigs. One gene, *clpP*, exhibited high sequence divergence in the first exon. To determine the full sequence of the gene, correctly identify the exon/intron boundaries, and confirm transcription of the gene, *clpP* was amplified from cDNA constructed from an individual from the same VA population. Another gene, *ycf1*, also exhibited high sequence divergence and appeared to have multiple frameshift mutations. However, these frameshift mutations were in long homopolymer or repetitive regions, raising the possibility they were due to 454 sequencing errors. PCR and Sanger sequencing confirmed that the frameshifts were the result of homopolymer-related sequencing errors. The corrected sequence yielded an intact *ycf1* reading frame. The final annotated contig sequences were deposited to GenBank under accession [GenBank:KJ920499-KJ920507].

### Interspecific divergence in cpDNA sequence

To estimate divergence and dN/dS ratios for plastid coding genes, the following species were used as outgroups and the corresponding gene sequences were obtained from GenBank: *Trachelium caeruleum* in the Campanulaceae [GenBank:NC_010442], and two more distantly related species, *Helianthus annuus* [GenBank:NC_007977] and *Nicotiana tabacum* [GenBank:NC_001879]. Outgroups were chosen to span a range of phylogenetic distances with one, *T. caeruleum*, in the Campanulaceae, and another, *H. annuus*, in the Asterales. Sequences were aligned with MUSCLE [23] as implemented in Codon Code Aligner v3.5 (CodonCode Corporation). High sequence divergence was observed for both *ycf1* and *ycf2*, necessitating the deletion of large regions of unalignable sequence. An average of 28 regions were removed per outgroup, with deletions averaging 78 bp and ranging up to 465 bp. Therefore, the resulting divergence values for these genes are underestimated. For *clpP*, the *T. caeruleum* sequence was re-annotated using DOGMA to locate the gene in the full chloroplast sequence obtained from GenBank and using homology between *C. americanum*, *H. annuus*, and *N. tabacum* to designate the intron/exon boundaries (Additional file 1). Gene alignments were deposited in Dryad [24].

The relative rates of sequence divergence and dN/dS ratio were determined for the protein coding genes using codon-based models of evolution in PAML v4.4 [25]. All analyses implemented a constrained topology with *T. caeruleum* and *C. americanum* monophyletic relative to *H. annuus* and *N. tabacum*, as *T. caeruleum* and *C. americanum* are within the same family. Codon frequencies were determined by an F3 × 4 model. The parameter values for dN/dS and transition/transversion ratio were estimated from the data with initial values of 0.4 and 2 respectively. Separate dN/dS values were estimated for each branch. Analyses were run on separate concatenations for each of the following sets of protein genes: 1) ATP synthase (*atp*), 2) NADH-plastoquinone oxidoreductase (*ndh*), 3) cytochrome b6/f complex (*pet*), 4) photosystem I (*psa*), 5) photosystem II (*psb)*, 6) large ribosomal subunit (*rpl)*, 7) small ribosomal subunit (*rps*), and 8) RNA polymerase (*rpo*), as well as the following individual protein genes: *ccsA*, *cemA*, *clpP*, *matK*, *rbcL*, *ycf1*, *ycf2*, *ycf3*, and *ycf4*. See Additional file 2: Table S1 for a list of specific genes included in each concatenation. The *psbT* gene was excluded from the analysis because it was multicopy in *C. americanum*, and *petN* was excluded because the *T. caeruleum* copy was unalignable. PAML estimated a dS value of zero for *clpP* on the terminal branch for *C. americanum*, resulting in an undefined dN/dS ratio. For subsequent analyses we estimated dS and dN/dS for *clpP* assuming a single synonymous substitution. All PAML files used were deposited in Dryad [24].

We tested for signatures of positive selection (defined by a dN/dS value significantly greater than one) by constraining the dN/dS ratio to one for the terminal branch leading to *C. americanum* for any genes where this branch had an initial estimated dN/dS ratio greater than one. Separate dN/dS values were estimated for each of the remaining branches. Likelihood ratio tests were used to compare the constrained and unconstrained analyses and determine if the estimated dN/dS ratios were significantly greater than one [26]. We applied a Bonferroni correction factor of 17 to account for multiple comparisons (17 genes/concatenations). To further examine sequence divergence and the potential for positive selection in one gene that showed a high dN/dS ratio, *clpP*, maximum likelihood trees were constructed for *clpP* intronic and exonic sequence separately using *Arabidopsis thaliana* as an outgroup. Trees were constructed using baseml in PAML v4.4 [25] with a fixed topology and a GTR model of evolution based on results from jModelTest v2.1.5 [27,28].

### Intraspecific polymorphism in cpDNA sequence

To identify within species polymorphism and estimate pN/pS (within species equivalent of dN/dS), 454 reads from the pooled multiple-population *C. americanum* sample were mapped to the assembled contigs from the VA sample using Roche’s GS Reference Mapper v2.5.3. The mapped reads had an average single copy coverage depth of 25×. A Perl script was written to use annotated gene locations and SNP information from the “HCDiff” mapping file to extract all high-confidence SNPs and identify them as genic/intergenic, exonic/intronic, and non-synonymous/synonymous (Additional file 3). Total numbers of non-synonymous and synonymous SNPs were tallied for each set of concatenated or individual genes, using the same concatenation groupings as used when estimating dN/dS. To estimate pN, pS, and the pN/pS ratio, the nonsynonymous and synonymous polymorphism counts were divided by the number of nonsynonymous and synonymous sites determined by PAML in our dN/dS analyses.

### Polymorphism and divergence

We determined whether similar sets of genes have an elevated dN/dS and pN/pS ratio by running a correlation analysis on the log transformed dN/dS and pN/pS ratios from each gene or concatenation (PROC CORR, SAS 9.3, SAS Institute, INC. 2011). Only genes or concatenations that had three or more SNPs were included in the correlation analysis (n = 11).

Additionally, to test whether genes with elevated dN/dS ratios have been under positive selection versus relaxed purifying selection, McDonald-Kreitman (M-K) tests [12] were run using Fisher’s exact test for each set of concatenated or individual genes (PROC FREQ, SAS 9.3, SAS Institute, INC. 2011). Pairwise divergence data between *C. americanum* and each of the three outgroup species (*T. caeruleum*, *H. annuus*, and *N. tabacum*) were obtained using a Perl script to extract all pairwise SNPs from the four species gene alignments used in the plastid divergence analysis above (Additional file 4). We applied a Bonferroni correction factor of 51 to account for multiple comparisons (17 genes/concatenations × 3 outgroup species).

## Results

### Plastid genome assembly

Sequencing of purified cpDNA from *C. americanum* produced high depth coverage of the plastid genome, which was assembled into nine contigs, totaling 147.3 kb. A complete assembly of *C. americanum’s* plastid genome was unattainable due to the presence of a large number of repetitive regions. These findings fit with those from the plastid genome of *T. caeruleum*, another member of the Campanulaceae, which also contains an unusually high level of repeats [29]. The plastid genomes of the Campanulaceae family have also been found to contain many inversions [13,14,29]. Likely this propensity for structural instability explains why mapping *C. americanum* reads to *T. caeruleum’s* plastid genome was not helpful in further assembly of *C. americanum’s* plastid genome. The potential for biparental inheritance of the plastid genome and subsequent heteroplasmy may have further contributed to difficulties in assembly when using a single maternal family.

Several genes commonly found in other angiosperms appear to be missing or presumed non-functional in *C. americanum*. No evidence was found for *accD*, even when searching against the raw reads, while *infA* is likely a pseudogene due to multiple internal stop codons. Only a 50 bp fragment remains of *rpl23*, though this fragment is present in at least two locations. The *rps16* intron has also been lost. These three genes and the *rps16* intron have also been lost or are presumed non-functional in *T. caeruleum* [29], suggesting these losses occurred prior to the divergence of these two species within the Campanulaceae. The loss of *accD* fits with evidence for this gene having been transferred to the nuclear genome in the Campanulaceae [30]. The *accD*, *rpl23* and *infA* genes have also been independently lost from the plastid genome in multiple other angiosperm lineages [31-33].

Several gene duplications have also occurred, which appear unique to *C. americanum*, though similar in pattern to those observed in *T. caeruleum* [29]. There has been a 300 bp partial duplication of *psbB* and a partial duplication of the beginning of *rrn16* upstream of the full *rrn16* gene. The *ndhF* gene has experienced multiple partial duplications including two identical 100 bp duplications as well as a separate 70 bp duplication. Several tRNAs were also duplicated, with two tandem copies of *trnM-CAU*, two copies of *trnS-GCU*, and three copies of both *trnfM-CAU* (two in tandem) and *trnL-CAA* spread throughout the genome. Duplications of tRNAs were also found in *T. caeruleum* where *trnI*-*CAU* is present in two copies [29].

One plastid gene, *psbT*, has undergone multiple duplication events, leading to three full-length copies in the *C. americanum* genome. One of these copies is highly conserved and retains the ancestral amino acid sequence when compared to *T. caeruleum*, *H. annuus*, and *N. tabacum*, while the other two copies have accumulated multiple amino acid changes. Again, a similar phenomenon was observed in *T. caeruleum*, where a different photosystem II gene, *psbJ,* is present in three copies [29].

### Interspecific divergence in cpDNA sequence

**Figure 1:**
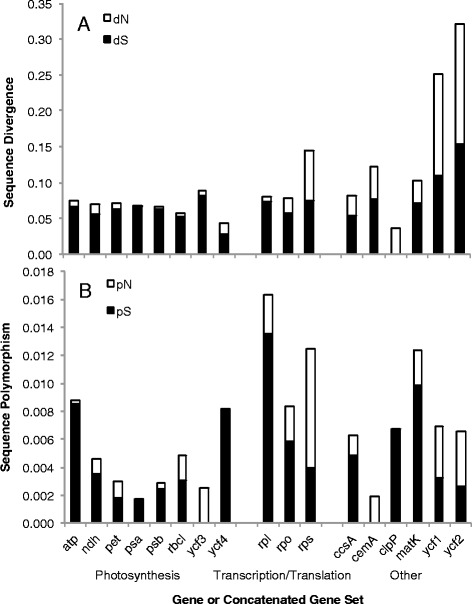
**Sequence divergence (A) and polymorphism (B) for** ***Campanulastrum americanum*** **protein coding plastid genes.** Sequence divergence and polymorphism as estimated by the number of substitutions per site in the terminal branch leading to *C. americanum* or within the species, respectively. Black and white bars indicate substitutions at synonymous and non-synonymous sites, respectively.

The dN/dS ratio varied widely across *C. americanum’s* plastid genes, suggesting these genes are experiencing different selective regimes (Figure 1, Table 2). The photosynthesis genes exhibited evidence of strong purifying selection, as indicated by their low dN/dS ratios (Table 2), while *ycf1*, *ycf2*, *clpP*, and the small subunit ribosomal protein genes had elevated dN/dS ratios close to or above one, suggesting the possibility of relaxed purifying selection, positive selection, or a mixture of both (Table 2, Figure 1). These genes also varied in the extent to which changes in dN or dS led to the elevated dN/dS ratios, suggesting that the selective regime leading to the elevated ratios may not be consistent across genes. Relative to other genes, *ycf1* and *ycf2* exhibited both an elevated dN and dS, while the small subunit ribosomal protein genes had only an elevated dN (Figure 1) The *clpP* gene exhibited a moderate increase in dN as well as a greatly reduced dS (Figure 1). The estimated dS of zero for *clpP* is likely to be a statistical anomaly due to the short sequence length providing a limited number of sites at which synonymous substitutions can occur. In addition, the branch leading to *T. caeruleum* and *C. americanum* exhibited an accelerated substitution rate and a dN/dS ratio greater than one for *clpP* (Additional file 5: Table S2). Therefore, the high dN/dS ratio found for *clpP* in *C. americanum* does not appear to be an artifact of the low estimated dS for this gene.

**Table 2:** **Sequence divergence and polymorphism values for** ***Campanulastrum americanum*** **protein coding plastid genes**

**Gene**	**dN/dS**	**pN/pS**	**# Polymorphisms**	**Gene Length**
Photosynthesis				
*atp*	0.131	0.031	11	4929
*ndh*	0.248	0.324	17	10263
*pet*	0.137	0.649	3	2289
*psa*	0.031	0.000	2	4929
*psb*	0.079	0.173	6	6360
*rbcl*	0.102	0.605	3	1425
*ycf3*	0.093	NA	1	504
*ycf4*	0.605	0.000	1	552
Transcription and Translation				
*rpl*	0.326	0.204	14	2757
*rpo*	0.354	0.428	33	10230
*rps*	0.915	2.135	36	4788
Other				
*ccsA*	0.509	0.290	2	918
*cemA*	0.578	NA	1	684
*clpP*	5.412* ^**a**^	0.000	1	555
*matK*	0.406	0.257	6	1491
*ycf1*	1.293	1.144	11	3042
*ycf2*	1.086	1.528	26	7137
All Genes	0.430^**b**^	0.518		

*Indicates significance before Bonferroni correction (p < 0.05). a: dN/dS ratio for *clpP* was estimated by calculating dN/dS as if there had been one synonymous SNP. b: mean dN/dS ratio was calculated without including *clpP*. NA: pN/pS ratio was inestimable due to pS being zero.

**Figure 2:**
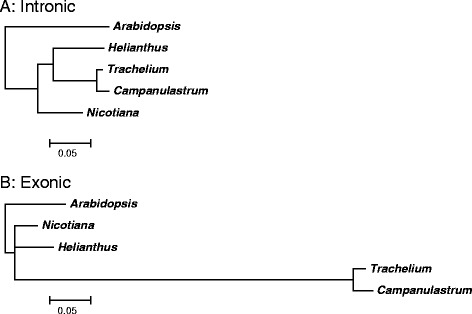
**Trees for** ***clpP*** **intronic (A) and exonic (B) sequence.** Trees with maximum likelihood branch length estimates using *Arabidopsis thaliana* as an outgroup.

The high dN/dS ratio suggests that positive selection may be acting on *clpP*. Further support for positive selection was identified when comparing branch lengths inferred from *clpP* intronic and exonic sequence. The exonic tree showed a greatly increased branch length (>7×) on the branch leading to *C. americanum* and *T. caeruleum* relative to the intronic tree (Figure 2). In contrast, the branch length on the branches leading to *H. annuus* and *N. tabacum* were shorter in the exonic tree relative to the intronic tree. These results are similar to those found in the tribe Sileneae (Caryophyllaceae), where evidence for positive selection on *clpP* was also observed when comparing intron and exon tree branch lengths [6]. The increased length and high dN/dS ratio (1.23) found for the branch leading to *C. americanum* and *T. caeruleum* suggest that altered selection was likely acting on *clpP* prior to the split of these two species (Additional file 5: Table S2).

### Intraspecific polymorphism in cpDNA sequence

We detected a total of 174 high confidence SNPs in 62853 bp of total protein coding sequence from the *C. americanum* plastid genome. As with divergence, the pN/pS ratio also varied strongly across plastid genes, suggesting that they are continuing to experience differing selective regimes at the within-species level (Figure 1, Table 2). Similar to the divergence results, the photosynthetic genes appear to be under purifying selection, while *ycf1*, *ycf2*, and the small subunit ribosomal genes had signatures of relaxed purifying or positive selection as evidenced by their elevated pN/pS ratios with values greater than one (Table 2, Figure 1). The elevated pN/pS ratios for these genes were primarily due to a higher pN, with the small subunit ribosomal genes in particular having a pN that is at least twice as high as for any other single gene or concatenation (Figure 1). On the other hand, *clpP* had a pN/pS ratio of zero, but that is based on only a single identified SNP in *C. americanum* (Figure 1, Table 2).

The patterns of polymorphism also suggested the existence of structural variation in the plastid genome within *C. americanum*. The initial polymorphism data indicated multiple non-synonymous SNPs within the first exon of *clpP*. Further examination of the pooled sequence data found evidence for a duplication of the first exon of *clpP* that did not appear to be present in the single population plastid assembly. Primers were then designed to amplify the first exon from either the full copy or duplication of *clpP*. The full copy first exon was amplified in all four populations used for sequencing the plastid genome (VA, AL, OH, and MN), while the duplication was only amplified in AL, OH, and MN, indicating the duplication does not exist in the VA population. These findings suggest the duplication event occurred since the divergence of the *C. americanum* populations. Sequencing and alignment of the partial duplication and full copy of *clpP* (deposited in Dryad [24]) recovered the nonsynonymous SNPs found in the initial polymorphism data, indicating they were artifacts caused by mapping the partial duplication to the full VA *clpP* copy.

### Polymorphism and divergence

**Figure 3:**
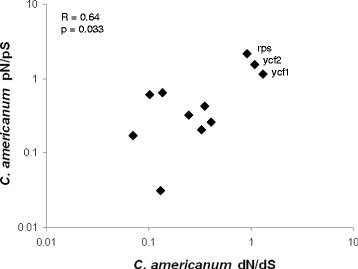
**Relationship between the dN/dS and pN/pS ratios for** ***Campanulastrum americanum*** **protein coding plastid genes.** Genes or concatenations with less than three SNPs, including *clpP*, are not shown. The three points with both an elevated dN/dS and pN/pS ratio are labeled.

Overall the dN/dS and pN/pS ratios are correlated across *C. americanum’s* plastid genes (Figure 3), suggesting that similar selective pressures are acting on the same genes across multiple time scales. The dN/dS ratios on the unrooted branch connecting *C. americanum* and *T. caeruleum* to *H. annuus* and *N. tabacum* are also correlated with *C. americanum’s* dN/dS ratios (R^2^ = 0.88, p < 0.001), further supporting similar selective pressures acting across time scales (Additional file 5: Table S2). While *clpP* seemed to deviate from this general pattern in that it showed a strongly elevated dN/dS ratio but a pN/pS ratio of zero, there is limited confidence in this estimate due to the short sequence length of *clpP* and the ratio being based only upon a single synonymous SNP.

In general, the M-K tests found no significant difference between the number of non-synonymous and synonymous SNPs when comparing the polymorphism and divergence data. The one exception was the set of the small subunit ribosomal proteins when using either *H. annuus* or *N. tabacum* as an outgroup. These analyses had a significant difference before, but not after, Bonferroni correction. In both of these comparisons, there was evidence for purifying selection, as the divergence among species had a lower ratio of non-synonymous to synonymous changes than the polymorphism within *C. americanum*. When using *T. caeruleum* as an outgroup, however, there was no longer a significant difference between the polymorphism and divergence SNP ratios for the small subunit ribosomal proteins.

## Discussion

Previous studies have found the Campanulaceae *sensu lato* to have relatively unstable plastid genomes characterized by a high frequency of inversions, the presence of repetitive regions, as well as gene duplications [13,14,29]. Fitting these earlier findings, assembly and annotation of *C. americanum’s* plastid genome revealed the presence of a number of repetitive regions as well as gene duplications. Further evidence for instability is suggested by a variable partial duplication of *clpP* among populations of *C. americanum*.

While the photosynthetic genes exhibited evidence for strong purifying selection, elevated nucleotide substitution rates and dN/dS ratios were found for *ycf1*, *ycf2*, *clpP*, and the small subunit ribosomal genes in *C. americanum*. The *ycf1* gene was recently found to be involved in protein translocation [34], while *ycf2* is essential for cell viability but of unknown function [35], and *clpP* codes for a protease subunit. The PAML analyses used average dN/dS across the entire gene (or gene concatenation), making this a conservative test for positive selection (dN/dS significantly greater than 1). If only a subset of codons within a gene/concatenation were under positive selection, the analyses used would have been unlikely to detect this signature. Accordingly, it is possible that some of the genes that exhibited evidence of purifying selection may have had positive selection at a subset of sites. At the same time, the genes with elevated average dN/dS ratios may have even higher dN/dS ratios at specific sites.

These genes with an elevated dN/dS ratio also differed in whether this was due to underlying changes in dN or dS, suggesting that the selective regime leading to the elevated ratios may not be consistent across genes. As synonymous substitutions are neutral, changes in dS are likely to reflect changes in the underlying mutation rate, potentially due to problems with DNA repair, whereas rates of non-synonymous substitutions are impacted not only by the underlying mutation rate, but also selection. Therefore changes in dN can also give insight into changes in selection. Relative to other genes, *ycf1* and *ycf2* exhibit both an elevated dN and dS, though the increase in dN was greater than dS (2.5× and 1.5× higher respectively), suggesting that the underlying mutation rate, as well as potentially the selective regime, has been altered in the these genes. In contrast, the small subunit ribosomal protein genes had only an elevated dN (Figure 1), strongly suggesting a change in selective regime, and not underlying mutation rate has led to the elevated dN/dS ratio in these genes. The *clpP* gene exhibits a moderate increase in dN as well as a greatly reduced dS. However, the uncertainties regarding the estimate of dS for this gene make it difficult to come to any conclusions regarding the underlying causes of the elevated dN/dS ratio.

A similar set of plastid genes (including *ycf1*, *ycf2*, *clpP*, and ribosomal protein genes) have increased nucleotide substitution rates and elevated dN/dS ratios in other taxa [3], including species within the tribe *Sileneae* [6,8,9], the genus *Oenothera* [6,7], and the Geraniaceae [4,10]. Increased nucleotide substitution rates have also been associated with increased structural variability [3], similar to our findings in *C. americanum.* These similarities suggest the possibility of a common evolutionary mechanism, whether adaptive or non-adaptive [9]. Our results suggest that this mechanism is continuing to operate at very recent time scales because we detect similar accelerated plastid evolution at the within-species level.

Almost all of the M-K tests were non-significant, indicating not only a lack of support for positive selection acting on the genes with an elevated dN/dS ratio, but also no support for purifying selection acting on the genes with a low dN/dS ratio. This lack of significance is probably due to the low power of the M-K tests we used, as a result of two factors. First, for several of the plastid genes/concatenations there are low levels of polymorphism (Table 2), which restricts the power of the M-K test. Second, one of the four *C. americanum* populations sequenced for this study (VA) has a plastid genome that is highly divergent from the other three. Therefore, most of the polymorphisms observed in *C. americanum* are likely fixed within populations and old enough that they have been subject to significant selection. The M-K test compares neutrally arising variation (within species polymorphism) to fixed differences after selection has acted (between species divergence) [12]. If selection has acted on within species polymorphism, this will reduce the power of the M-K test to detect a significant difference between the polymorphism and divergence data. Therefore, when there is substantial intraspecific divergence, such as in *C. americanum*, the nature of the polymorphism data makes it difficult to definitively distinguish between the contributions of positive selection and relaxed purifying selection to the increased nucleotide substitution rates and dN/dS ratios observed in some plastid genes. Future studies using increased sampling could allow for more sensitive tests for positive selection. For example, the recent sequencing of dozens of chloroplast genomes within the Campanulaceae *sensu lato* [14] raises the future possibility of using phylogenetic tests to look for site-specific positive selection.

Accelerated plastid evolution may be an important contributor to the development of reproductive isolation and subsequent speciation. The nuclear and plastid genome are likely to be co-evolved as they must interact closely to carry out essential functions, such as photosynthesis [36]. Increased nucleotide substitution rates, altered selective regimes, and increased structural variation have the potential to lead to rapid local co-evolution of these genomes, leading to an increased likelihood for cytonuclear genetic incompatibilities when crossing between populations. Cytonuclear incompatibilities are proposed to be among the first genetic incompatibilities to arise [37,38] and are increasingly thought to play an important role in the speciation process as they have been implicated in contributing to reproductive isolation in plants, yeast, and animals [39-42].

**Figure 4:**
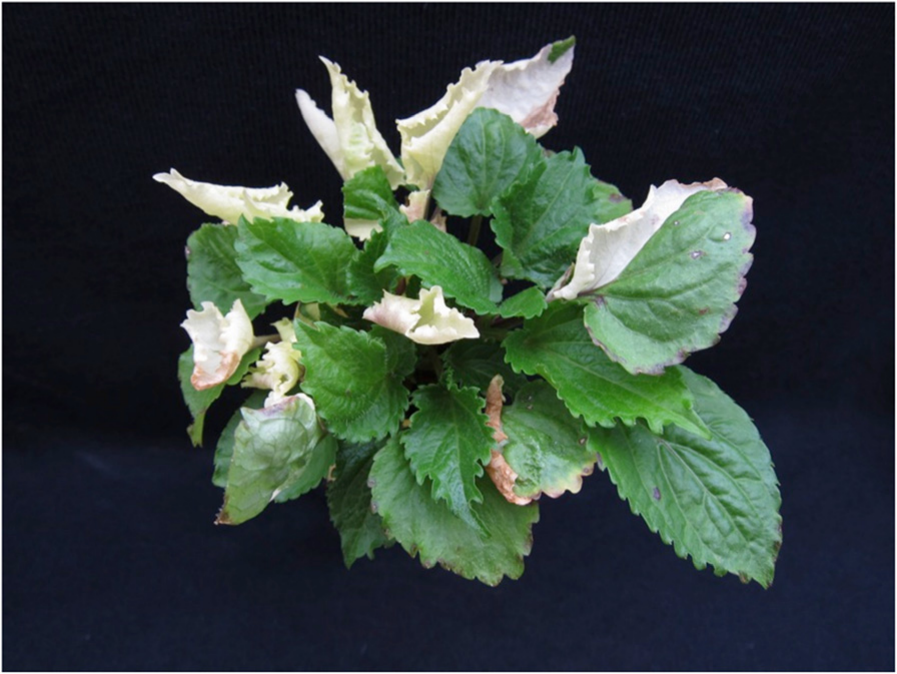
**Intraspecific first-generation (F1) hybrid of** ***Campanulastrum americanum*** **showing variegation.** This variegation is representative of what is found when crossing between populations with high levels of genetic divergence, such as the divergence between Virginia and remaining three populations used in this study.

Strong reproductive isolation is found between populations of *C. americanum,* and the asymmetrical pattern of this breakdown along with observations of chlorosis and variegation (Figure 4) suggest cytonuclear incompatibilities contribute to this isolation [15,43]. Positive and or relaxed purifying selection on *ycf1*, *ycf2*, *clpP* and the small subunit ribosomal genes, as well as the general instability of the plastid genome, may contribute to cytonuclear incompatibility and reproductive isolation in *C. americanum*. The small subunit ribosomal genes in particular are interesting candidates for intraspecific cytonuclear incompatibilities due to their highly elevated level of pN and the fact that nuclear-encoded subunits of organelle ribosomes have been found to exhibit evidence of compensatory substitutions in response to rapid evolution of organelle genomes [44,45].

Similar to *C. americanum*, other independent lineages of angiosperms have increases in nucleotide substitution rate and elevated dN/dS for a subset of plastid genes [1,3]. Could accelerated plastid genome evolution also contribute to cytonuclear incompatibility and reproductive isolation in these lineages? Cytonuclear incompatibilities are known from interspecific crosses in *Oenothera* [reviewed in [46]] and in *Pelargonium* [47,48], both of which exhibit similar patterns of accelerated plastid evolution. However, cytonuclear incompatibilities and reproductive isolation are rarely examined at the intraspecific level. Further work in examining the relationship between accelerated plastid evolution, cytonuclear incompatibility, and reproductive isolation at the intraspecific level would allow for a more general conclusion as to whether accelerated evolution and positive selection on plastid genes could help drive the early stages of the speciation process.

## Conclusions

We found increased nucleotide substitution rates when examining intraspecific polymorphism in the plastid genome of *C. americanum*. In addition, there was a significant correlation between the dN/dS and pN/pS ratios across plastid genes. These results provide evidence that mechanisms leading to increased nucleotide substitution rates in the plastid genome are continuing to operate at recent evolutionary timescales and may, therefore, be contributing to the early stages of the speciation process through the development of intraspecific cytonuclear incompatibilities and reproductive isolation.

## Availability of supporting data

The data sets supporting the results of this article are available in the Dryad repository, [doi:10.5061/dryad.d143r, http://datadryad.org/resource/doi:10.5061/dryad.d143r].

## Additional files

Additional file 1:
**Sequence of** ***clpP*** **for** ***Trachelium caeruleum*** **with re-annotated intron/exon boundaries.** Exons in capital letters and introns in lowercase. Additional file 2: Table S1. Designation of which individual genes were included in concatenations. Also includes list of genes analyzed individually. NA: genes excluded from analysis, see text for details. Additional file 3:
**Perl script used for extracting intraspecific SNPs.**
Additional file 4:
**Perl script used for extracting interspecific SNPs.**
Additional file 5: Table S2. dN/dS ratios for all four species used in the divergence analyses. dN/dS ratios from the codeml analyses for the terminal branches leading to each species as well as for the internal branch leading to the common ancestor of *T. caeruleum* and *C. americanum*. *: *N. tabacum* is the outgroup in the analysis, and therefore the reported values are a combination of the terminal branch and the divergence on the internal branch leading to the Asterales. a: dN/dS ratio for *clpP* was estimated by calculating dN/dS as if there had been one synonymous SNP.
