# Supplementary material for: Correlation between sequence divergence and polymorphism reveals similar evolutionary mechanisms acting across multiple timescales in a rapidly evolving plastid genome
Source: BMC Evol Biol. 2014 Dec 24;14:1. doi: 10.1186/s12862-014-0268-y (PMC4300152; doi:10.1186/s12862-014-0268-y)
Supplement: Additional file 1: — Sequence of clpP for Trachelium caeruleum with re-annotated intron/exon boundaries. Exons in capital letters and introns in lowercase. [file 12862_2014_268_MOESM1_ESM.pdf]

Additional file 1.pdf: Sequence of *clpP* for *Trachelium caeruleum* with re-annotated intron/exon boundaries. Exons in capital letters and introns in lowercase.

ATGCCAGTTGGTGTTCCGAAAGTCCCACCTATGCCACTTGAACCTGAATCTGAAAA
AAATCAGAAAAAGAAACCTAAACAAGGATTCCTCGAAAAATCCCTCAAATTCCACAA
AAGCCTAGCACGAGAAAAAGAATTCATAGACGAAGAAGAAGTAGATGAAGAAGAAG
TAGATGAAGAAGAAGTAGATGAAGTATATGAAGTATATGAAGAAGAAGTAGAAAAAG
TAGAAACAAAAAAAAAAGGAAAAAGACGGAGAAGAAGAGGAGGAACAAGAGGAGGA
AGAGGAACCTTGGGTTGACTTATAgtgcgacttgtcagatatattggctcatatgggatttccccgttctctccc
cgattgagagatcctctatttcgcccaagaaagattaattgaatcatccaaaatttggagcgtgaagtccaattagatacatttt
ttaggggcattcaaattactattagccgttcgaataatttatggttggcttggttggacgaataaattgaagtatccaggctccgt
ttaaaaaaaccaattggtaatctatctaccattactccttatatcataaacggattcctagttagaaagaaatcttatttgtaaata
gaaatgatctgaaactgttctgttaatcaatcgagtaatatgcatgaagacctggaatatttgccgaaatgcctgaattgaga
aaaaagaagtggtaatgggagtatttgttctatatgtgcaaatcaaaagcgggtggatctttacccggagtagagtataaac
ctaaaaagattcagattaacgaggtccatttaggaacaagcaaatgacatcgtgatttgaattggatctcgatgaaagacta
tatcaatgaaaagtgaattcgataagtttcattaattctttactcgtctttattgaaaatcgaatcaaaatgagaagtccgaaag
agcattctatgaaatccgaaaggggattggaatctatacattgattttttcgcaaaattttggaaccgtatgcgtcaaaaggc
gcctgtacggttcctaaggaatagaatttgaccctaatCGAACGACTTTATCGAGACAGAGCACTTTTT
TTATTCAAAGAGCTGGATAAGGAGCTCGCGAATACACTTGTGGGTCTTATGGTATTT
CTCAATATAGAGGATAATACCAAAGAACAATTTTTATTTATCAACTCTCCTGGTGGAT
CACTAGTGTATGGAATATCTGTGCATGATGTTAGCCGACTGGTGCGACCAGATGTA
CATACACTAGGCATGGGAGTAGCCGCTTCAATGGCCGCTTTCATCCTGTCCGGAG
GAGCACAAACCAAACGTCTAGCATTCCCTCACGTTtggcgccaatgaggttttatttcagagaaaaa
agaagactatgccttcgccatatgaaatatgaatagtaagtaatataagtaataatagcatggcactttgaattcgatatatga
aagtttttgattgtttttcaaagcattagattatgtatcgagagagtagtatgagataaaaggtatttatgatttttcttatctatcgg
gagtccagttcagcgtcacaaactttttgctttcccaccggagatctagtaacaatatttatgttatgaacgagtgaaaaaaa
aaaaaaaaaaaaaagattctttttccttagtttatttaatcaaataaaaaagcaactttgggattgcttaatcatagacaaaaa
aagaaatccatatataaagcaacggagccatcatagtagttttgaactcccacgaaaggaagggtggtaatttgatcattta
ctgatcgaggtctaatagatcctatttttatctttctttgatagagggtaaggatcaatttgattgtagagccgtatgcaatgcaca
aaagatgcctgtacggttgttcaattctatcttttttcttcttattcttttatctttctttcttttatcttccctttatcaggcgaactagaaga
accttttattatatcatcAGGGTAATGATTCATCAACCAAAATGTGTTTTTGCAAAGAATCGCAT
CCCGATTGACGTAGGCCTGGACGGAGAAGAAGTGACAAAATTACGTAACTACGTCA
TAGGACATTATGCACAAAGATCGCGCAGGTCTATAGCGATGGTAGTCGCCGACCTG
AAAAGACATACTTATATGACACCAACAGAGGCCCGAACTTATGGAATTGTTGATTCT
ATAGCGGCTGACTGGGAGGTCTACTAAATCCATGATTTGA
